# Reduction of psychological cravings and anxiety in women compulsorily isolated for detoxification using autonomous sensory meridian response (ASMR)

**DOI:** 10.1002/brb3.2636

**Published:** 2022-06-08

**Authors:** Mei Qi Hu, Hui Ling Li, Si Qi Huang, Yu Tong Jin, Song Song Wang, Liang Ying, Yuan Yuan Qi, Xin Yu, Qiang Zhou

**Affiliations:** ^1^ Department of Psychology Wenzhou Medical University Wenzhou China; ^2^ The Affiliated Kangning Hospital Wenzhou Medical University Wenzhou China; ^3^ Renji College Wenzhou Medical University Wenzhou China; ^4^ Zhejiang Moganshan Female Drug Detoxification Center Huzhou China

**Keywords:** autonomous sensory meridian responses (ASMR), detoxification, psychological cravings, forced abstainers, anxiety

## Abstract

**Objective:**

To explore the effects of the autonomous sensory meridian response (ASMR) on the psychological cravings and anxiety of women compulsorily isolated for detoxification.

**Method:**

Around 122 women were recruited in a female drug detoxification center. Except for the 12‐week training of ASMR, the experimental conditions of the experimental group (*n* = 60) were the same as those of the control group (*n* = 62). The addiction Stroop task was used to assess the level of psychological cravings and the State‐Trait Anxiety Inventory was used to assess the level of anxiety.

**Results:**

After the training, the decrease in state anxiety of the experimental group was larger than that of the control group, and the reaction time of the experimental group in the Stroop was also significantly lower than before the training.

**Conclusions:**

ASMR could thus reduce to a certain extent the state anxiety and attentional bias for drug‐related clues under signaling psychological cravings among women compulsorily isolated for detoxification.

**HIGHLIGHTS:**

- Intervention effects on psychological cravings and anxiety of women isolated for detoxification
- Basis for role of ASMR in regulating psychological cravings and anxiety in forced abstainers
- ASMR intervention reduced forced abstainers’ attentional bias to drug‐related clues

As a global social issue, the still severe problem of drug addiction poses a serious threat to human health and social development, given the issues of extreme dependence and relapse rate. In current detoxification work, there are two commonly used models for drug addiction: One is a physical and medical rehabilitation model based on drug substitution therapy, and the other is a social psychological rehabilitation model based on psychological intervention and cognitive behavior modification (Lin et al., 2021). In China, compulsory isolation for detoxification is the mainstay of treatment, and various compulsory, punitive, educational, and corrective technical methods are used to help drug users overcome addiction.

Among these methods, psychological cravings are particularly challenging in the context of abstinence. From the perspective of psychological research, psychological cravings refers to an addict's uncontrollable impulsive desire for the past subjective experience of psychoactive substances; and operationally, it can be defined as the psychological preference and implicit attitude derived from the long‐term addictive behavior of the addict, which is difficult to control (American Psychiatric Association, 2013). Many factors affect psychological cravings. Among them, addicts’ negative emotions are beginning to attract increasingly more attention and research interest. Susceptibility factors such as anxiety, depression, and fear play a key role in the craving for addictive substances, and to a certain extent, can positively predict or assess relapse in addicts (Baker et al., 2004; Pani et al., 2010; Zhou et al., 2019).

It is well known that men predominate among traditional drug abstainers, but methamphetamine (MA) abstainers are similarly divided between men and women (Durell et al., 2008). Previous studies, however, have focused more on MA male abstainers (Cui et al., 2021;Gao et al., 2022; Li et al., 2022; Zhao et al., 2021). It is worth noting that the mental health problems of women undergoing compulsory drug rehabilitation are more serious than those of men in China (Wen and Li, 2007; Xia, 2015). Female MA abstainers exhibit more severe psychiatric symptoms than men, and they seek treatment for major depression and suicidal ideation more commonly than men (Darke et al., 2011). In a study, a significantly higher proportion of women with MA withdrawal met criteria for anxiety disorders than men (Glasner‐Edwards et al., 2010). Notably, the intensity of craving in MA withdrawal is positively correlated with the scores obtained from the Symptoms Checklist‐90 (Nakama et al., 2008), and craving in MA withdrawal is strongly correlated with anxiety, and they are often accompanied by mood disorders such as anxiety disorders, which is the starting point for developing withdrawal treatments (Hartwell et al., 2016), which suggests the need to consider the level of psychological cravings and anxiety among women undergoing compulsory drug rehabilitation.

Further, numerous studies on the attentional bias of drug addicts have found that psychological cravings can cause addicts to skew their attention—subconscious overattention is paid to cues related to addictive substances (Field et al., 2014; Witkiewitz and Bowen, 2010). This, in turn, causes craving and creates a vicious circle. The addiction Stroop task and dot‐probe task are two commonly used research paradigms to assess attentional bias. Compared with the dot‐probe task, the improved addiction Stroop task has higher internal consistency, thus being more suitable to study the attentional bias of addicts related to substances (Ataya et al., 2012). Simultaneously, the study of Cao, Sun, and Deng (2015) also fully confirmed a significant association between performance in the addiction Stroop task and psychological cravings. That is, the higher the addicts’ attentional bias toward drug‐related cues, the worse their Stroop task performance, and the higher their level of psychological cravings. This suggests that the Stroop interference effect can be an indirect measure of the level of psychological cravings.

Recently, media related to the autonomous sensory meridian response (ASMR) have frequently appeared on Internet platforms such as Reddit ASMR forum and the video site YouTube. ASMR is an atypical physical–psychological experience with a dynamic, wave‐like, static‐like tingling sensation, triggered by a specific audio–visual stimulus. It is also called “intracranial tingling” or “intracranial orgasm.” It usually originates from the back of the scalp and gradually progresses along the spine to the shoulders or limbs, accompanied by positive feelings such as relaxation, happiness, euphoria, and elevated mood. Common triggers include watching someone whisper, performing repetitive rhythmic movements, and exploring an object; however, the coverage of the tingling seems to depend on the degree to which the individual is triggered (Barratt and Davis, 2015). At present, the exploration of the physio‐psychological mechanism behind ASMR is still in the early stage, and mainly focuses on the neurobasic research and case report research on the causes of positive sensory emotions, attention, improvement of pain, and social cognition (Reddy and Mohabbat, 2020). But public interest in it is growing, and more researchers are actively seeking to use it as a complementary therapy for appropriate treatment.

In the first assessment of ASMR, Barratt and Davis (2015) found that ASMR participants reported a temporary relief of chronic pain and mood improvement. Among them, 80% of the participants reported that ASMR had positive emotional effects (relaxation and euphoria). Simultaneously, ASMR had a “placebo effect”—the first contact with ASMR media may cause a somatosensory response that meets personal expectations, while more frequent users usually experienced a sense of relaxation and satisfaction actually triggered by ASMR media (Cash et al., 2018). Therefore, the sensitivity to ASMR may also influence its effects.

Given the positive effects of ASMR on mental health and emotional regulation (such as relieving stress, anxiety, loneliness, and insomnia), ASMR‐related academic studies have gradually become popular in recent years. ASMR has been associated with other topics and technologies in exploratory research. However, there are no literature reports on the application of ASMR to forced abstainers. Therefore, this study pioneers the application ASMR to the regulation of anxiety in forced abstainers, especially the psychological cravings in the context of abstinence.

We propose that ASMR can reduce anxiety effectively and shift the addicts’ attentional bias from drug‐related cues progressively to eliminate the psychological cravings for drugs in the withdrawal response of forced abstainers. Besides, we also propose that ASMR sensitivity characteristics may influence the effectiveness of the emerging methods. Thus, ASMR is expected to be employed as a specialized treatment tool and ultimately help addicts to achieve the recovery of physical and mental health and return to normal social life at the earliest.

## 1 METHODS

### 1.1 Research design

A randomized controlled trial was conducted, with a three‐factor mixed design of 2 (ASMR training/no training) × 2 (ASMR sensitive/nonsensitive) × 2 (ASMR with semantic dialog/without semantic dialog).

This study was approved by the Ethics Committee of a medical university (Approval no. 2020–122). All participants voluntarily participated in the study and signed an informed consent form. Participants could withdraw at any time if they were unwilling or unable to continue with their participation in the study.

### 1.2 Participants

According to the sample size calculation from G‐Power, 128 participants were required (*f* = 0.25, 1‐*β* = 0.8), and 122 were finally included in the analysis. First, a preliminary screening was performed using the following eligibility criteria: (1) women over 18 years of age; (2) meeting the Chinese classification of mental disorders (CCMD‐3) diagnostic criteria for drug dependence and having no dependence on other psychoactive substances except new drugs; (3) right‐handedness; (4) normal or corrected vision and normal or corrected hearing; (5) no use of psychiatric medicines within 2 weeks; and (6) no history of neuropsychiatric diseases and no infectious diseases(See Appendix A for more details on other clinic information). Complementally, compared with traditional drugs such as opium and heroin, in China, new drugs mainly refer to psychoactive substances of artificial chemical synthesis, which directly act on the central nervous system and lead to the mental dependence or substance abuse with continuous use of these drugs. It is also controlled by international antidrug conventions and Chinese laws and regulations.

During the training period, participants were required not to participate in other psychological or behavioral treatments. A total of 193 of 650 screened women fulfilled the eligibility criteria and were not arranged to leave the drug detoxification center.

**FIGURE 1:**
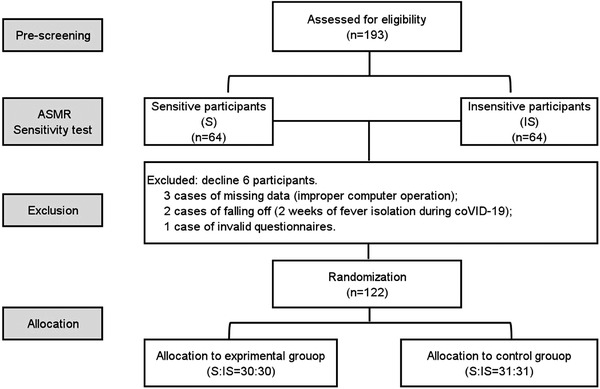
Flow diagram of participants’ screening and allocating. In addition to the ASMR intervention training in the experimental group, the other experimental settings were consistent with the control group

Subsequently, through a self‐report survey of ASMR sensitivity, 64 sensitive participants (S) to ASMR videos were selected from the 193 eligible participants, and 64 insensitive participants (IS) were randomly selected from the remaining participants. During the ASMR training period, three participants had missing data (improper operation on the computer), two participants dropped out (isolated for 2 weeks due to fever during the COVID‐19 epidemic), and one filled out invalid questionnaires multiple times. After excluding these participants, data from 122 participants were finally included in the analysis, including 60 in the ASMR group (S:IS = 30:30) and 62 in the control group (S:IS = 31:31) the balance between the groups with ASMR sensitivity characteristics. (See Figure 1).

### 1.3 Materials

#### 1.3.1 ASMR videos

The foremost videos containing ASMR triggers from the ASMR video library compiled by Liu and Zhou (2019) were selected, comprising 59 videos (i.e., 30 ASMR videos with semantic dialog and 29 ASMR videos without semantic dialog, produced by performers of different genders). The length of each video was between one and three minutes. (See Appendix B and C for details on the material).

#### 1.3.2 Psychological cravings

The addiction task under the Stroop paradigm was used to determine the participants’ level of psychological cravings. The stimulus material included 30 drug‐related Chinese characters and 30 neutral Chinese characters. After randomized sorting using Excel, manual sorting was performed to make the Chinese characters of different nature appear pseudo‐randomly in three colors—red, yellow, and green. This was done to avoid the continuous presence of Chinese characters of the same color or the same nature for up to three times in a row (Cao et al., 2015). Besides, the interference effect of Stroop was evaluated mainly through accuracy rate and reaction time. (See Appendix D for details on the material).

#### 1.3.3 State‐trait anxiety

The State‐Trait Anxiety Inventory (STAI) compiled by Spielberger et al. (1983) was used, which comprises a total of 40 items divided into two subscales—State Anxiety Inventory (SAI) and Trait Anxiety Inventory (TAI)—each with 20 items. Items are scored on a 4‐point Likert‐type scale, and all positive emotions were scored reversely (9 items in SAI and 10 items in TAI). The scores on each subscale range from 20 to 80, and higher scores indicate higher state or trait anxiety levels. In this study, we used the Chinese version of the STAI, and its reliability and validity have been verified in previous studies (Shek, 1993).

#### 1.3.4 Procedure

The control group only completed the TAI and addiction Stroop tasks at pretest and posttest and the SAI with the same frequency as the experimental group's training period. On this basis, the experimental group completed periodic ASMR training (twice a week, at an interval of 2–3 days, 24 times over 12 weeks in total), and the training time lasted from July 5 to September 25, 2020. The ASMR group completed the SAI assessment immediately after the completion of ASMR training, while the control group completed the SAI assessment within the same day and period.

The addiction Stroop tasks were performed using PsychoPy 3, including two stages: Practice and Stroop‐test. The test stage contains three blocks, and there were 30 drug‐related Chinese characters and 30 neutral Chinese characters that appeared pseudo‐randomly in each block. The subjects were asked to select one of the three keys as quickly and correctly as possible according to the color of the Chinese characters in the picture. The different keys represented red, blue and green colors, respectively. There was a break of 2–3 min between each test group. And the procedure automatically recorded the response time and accuracy of the key to the picture within 3000 ms.

The ASMR training (watching three ASMR videos of different intensity and content) was always conducted in the same computer room in the drug detoxification center. During the training, the conditions were as follows: (1) the environment was quiet and undisturbed, and appropriate activity space was reserved for the participants; (2) the headset functions were intact, and the volume was adjusted to 80% and the screen brightness to 50% uniformly; (3) the ASMR video was played according to the instructions.

### 1.4 Statistical analysis

All statistical analyses were performed using IBM SPSS 23.0. For some raw data involving the privacy of the participants, appropriate conversions were performed first; the changes do not affect the actual statistics. All descriptive statistics were provided with 95% confidence intervals across participants. Student's *t*‐test was used to evaluate the association between ASMR intervention and, attentional bias, or state anxiety, respectively. Three‐factor repeated‐measure ANOVA was used for the interaction effect among sensitivity, semantic dialog, and training period on attentional bias and state anxiety. For all comparisons, *p* < 0.05 was considered statistically significant.

## 2 RESULTS

### 2.1 Demographic characteristics

The age range of the participants was 18–54 years, with an average age of 33.83 years (SD = 8.612); participants were mostly young. In terms of education level, most of them had a junior high school degree (*n* = 78, 63.93%), and the average education level was low. Moreover, all participants had undergone detoxification not more than three times. The addictive substances they used were mainly excitatory synthetic drugs with MA as the main component.

There were no significant differences between the two groups in age, education level, frequency of detoxification, type of addiction, employment status, or income level (*p* > 0.05). However, in terms of family and marriage, more participants in the experimental group experienced failed marriages (40.0%) or had children (68.3%) (*p* < 0.05); more participants in the control group were still single (56.5%) or, childless and not pregnant (66.1%) (*p* < 0.05). (See Table 1).

**TABLE 1:** Baseline demographic characteristics of 122 participants

Participant Characteristics	Experimental Group	Control Group	*T*
	(*n* = 60)	(*n* = 62)	
Age groups (years), *n* (%)	*M* 35.30 ± *SD* 8.821	*M* 32.40 ± *SD* 8.225	1.877
18–29	16 (26.7)	25 (40.3)	
30–39	24 (40.0)	23 (37.1)	
40≤	20 (33.3)	14 (22.6)	
Marital status, *n* (%)			2.650^**^
Single	20 (33.3)	35 (56.5)	
Married/Cohabited	16 (26.7)	13 (21.0)	
Divorced/Separated/Widowed	24 (40.0)	14 (22.6)	
Fertility status, *n* (% Nullipara)	19 (31.7)	41 (66.1)	4.022^***^
Education levels, *n* (%)			‐1.640
Primary or below	9 (15.0)	6 (9.7)	
Junior high	41 (68.3)	37 (59.7)	
Senior high	6 (10.0)	13 (21.0)	
Graduate or above	4 (6.7)	6 (9.7)	
Frequency of compulsory isolation and detoxification, *M* ± *SD*	1.23 ± 0.465	1.27 ± 0.548	‐0.443
Types of substance addiction, *n* (% Methamphetamine)	59 (98.3)	60 (96.8)	‐0.552
Employment status ^a^, *n* (% Unemployment)	37 (61.7)	38 (61.3)	‐0.042
Monthly personal income level ^a^, *n* (%)			0.967
None	28 (46.7)	36 (58.1)	
≤¥2,200	2 (3.3)	3 (4.8)	
(¥2,200, ¥5,000]	14 (23.3)	11 (17.7)	
(¥5,000, ¥10,000]	12 (20.0)	4 (6.5)	
¥10,000≤	4 (6.7)	8 (12.9)	

### 2.2 Psychological cravings

#### 2.2.1 Intergroup effect of ASMR on attentional bias

In the intragroup analysis of differences in the addiction Stroop task performance, there was no significant difference in accuracy (Acc) comparing before and after the operation for the experimental or the control group (*p* = 0.525, *p* = 0.343). However, the reaction time (Rt) of the experimental group was significantly reduced after the training compared to before (*p* = 0.01), while the control group showed no significant difference (*p* = 0.946).

In the analysis of differences between groups, there was no significant difference in Acc or Rt between the two groups in the pretest (*p* > 0.05). After the training, there were no significant differences between the groups in Acc. However, the experimental group had a lower reaction time than the control group, and the difference was significant (*p* < 0.05). (See Table 2).

**TABLE 2:** In the Stroop task of addiction, the difference analysis of attention bias between groups

	Pre‐test (95% *CI*)			Post‐test (95% *CI*)		
	Experimental Group (*n* = 60)	Control Group (*n* = 62)		Experimental Group (*n* = 60)	Control Group (*n* = 62)	
	*M* ± *SD*	*M* ± *SD*	*t*	*M* ± *SD*	*M* ± *SD*	*t*
Stroop task						
Acc (%)	92.82±13.44	95.39±5.16	‐1.398	94.22±12.58	95.29±11.55	‐0.487
Rt (ms)	848.72±205.08	841.06±204.71	0.206	763.71±130.17	820.70±178.62	2.019*

Abbreviations: M, mean; *SD*, standard deviation; *CI*, confidence interval; Acc, accuracy; Rt, response time.

* *p* < 0.05.

#### 2.2.2 Intragroup effect of sensitivity, semantic dialog, and training period on attentional bias

The results of three‐factor repeated‐measure ANOVA on attentional bias showed that, for Acc and Rt, the main effect of ASMR sensitivity (*F*_(1,13)_ = 2.263, *p* > 0.05, *η* ^2^ = 0.148; *F*_(1,13)_ = 0.978, *p* > 0.05, *η* ^2^ = 0.070), semantic dialog (*F*_(1,13)_ = 0.765, *p* > 0.05, *η* ^2^ = 0.056; *F*_(1,13)_ = 0.550, *p* > 0.05, *η* ^2^ = 0.041), or training period (*F*_(1,13)_ = 0.270, *p* > 0.05, *η* ^2^ = 0.020; *F*_(1,13)_ = 0.472, *p* > 0.05, *η* ^2^ = 0.041) was not significant. And none of interactions was significant either.

### 2.3 Anxiety

#### 2.3.1 Intergroup effect of ASMR on state anxiety

Before the training, there was no significant difference in the trait anxiety (TAI scores) between the experimental and control groups (*p* = 0.078). That is, the individual differences (trait anxiety, TA) were consistent in the anxiety level of the participants, which made the time dimension (state anxiety, SA) comparable between the groups.

**FIGURE 2:**
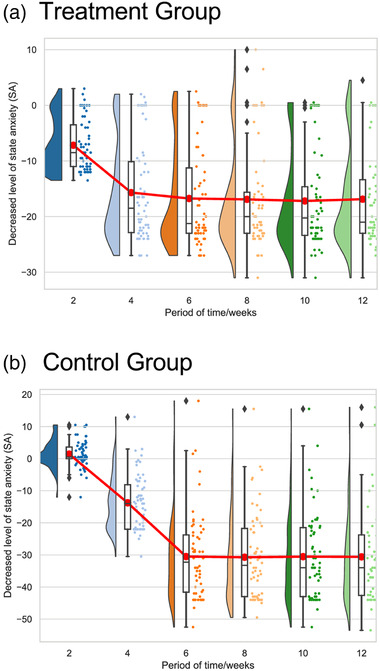
Changes in the decreased level of state anxiety (SA) between groups during the training period (*M* ± *SE*): compared with pretest, the change of the experimental group was significantly after six weeks of the training (*p* < 0.01)

In the analysis of differences at different points of the training, the SAI scores of the two groups were not significantly different when the training had been conducted out for 1 month. After 6 weeks of the training, the SAI score of the experimental group was significantly lower than that of pretest (8 weeks: *M* = −13.684±*SD* 2.039; 12 weeks: *M* = −13.577 ± *SD* 2.079) (*p* < 0.01). That is, the training effect after 6 weeks in SA reduction in the experimental group was larger than in the control group. (See Figure 2).

### 2.4 Intragroup effect of sensitivity, semantic dialog, and training period on state anxiety

The results of three‐factor repeated‐measure ANOVA on state anxiety showed that the main effect of ASMR sensitivity was not significant, and there was no significant difference in the decreased degree of state anxiety between ASMR sensitive participants (*M* = ‐21.171±*SD* 1.910) and ASMR nonsensitive participants (*M* = ‐25.088±*SD* 1.947), *F*_(1,13)_ = 2.165, *p* > 0.05, *η* ^2^ = 0.143.

Besides, the main effect of semantic dialog in ASMR was significant. Compared with nonsemantic dialog (*M* = ‐26.856±*SD* 1.900), the decrease of state anxiety level was significantly lower, *F*_(1,13)_ = 6.080, *p* = 0.028, *η* ^2^ = 0.319.

Also, the main effect of ASMR training period was also significant, *F*_(1.566, 20.362)_ = 302.092, *p* < 0.01, *η* ^2^ = 0.959. Specifically, after 6 weeks of training (*M* = ‐30.040±*SD* 1.641), compared with 2 weeks *(M* = 2.299±*SD* 0.536) and 4 weeks after training (*M* = ‐21.045±*SD* 1.233), the level of state anxiety decreased significantly (*p* < 0.01). While in the following weeks of training, there was no significant difference in the decrease in state anxiety levels (8 weeks: *M* = ‐29.875±*SD* 1.645; 10 weeks: *M* = ‐30.009±*SD* 2.043; 12 weeks: *M* = ‐30.107±*SD* 1.757)(*p* > 0.05).

In the pair interaction analysis, ASMR sensitivity and ASMR semantic dialog, ASMR semantic dialog and ASMR training period both had no significant interaction, *F*_(1,13)_ = 2.481, *p* > 0.05, *η* ^2^ = 0.160; *F*_(1.368, 17,780)_ = 2.601, *p* > 0.05, *η* ^2^ = 0.167. However, the interaction between ASMR sensitivity and ASMR training period was significant, *F*_(1.579, 20.533)_ = 4.008, *p* = 0.042, *η* ^2^ = 0.236.

In addition, ASMR sensitivity, ASMR semantic dialog, and ASMR training period had significant interaction, *F*_(1.730, 22.496)_ = 3.677, *p* = 0.047, *η* ^2^ = 0.220. In the further analysis of simple effect, under ASMR sensitive and semantic dialog condition, the level of state anxiety decreased significantly after 6 weeks of training (*M* = ‐33.893±*SD* 2.696) compared with the previous couple of weeks (2 weeks: *M* = 0.250±*SD* 1.499; 4 weeks: *M* = ‐24.536±*SD* 2.435)(*p* < 0.01). While the decreased degree of state anxiety level during the following weeks of training was not significantly different from that after 6 weeks (8 weeks: *M* = −34.571±*SD* 2.494; 10 weeks: *M* = −34.143±*SD* 2.820; 12 weeks: *M* = −34.893±*SD* 2.680)(*p* > 0.05).

Under ASMR sensitive and no semantic dialog condition, the decreased degree of state anxiety level after 4 weeks (*M* = −14.339±*SD* 3.143) was significantly lower than that after 2 weeks (*M* = 2.179±*SD* 1.196) (*p* < 0.05). While the decreased degree of state anxiety level during the following weeks of training was not significantly different from that after 4 weeks (6 weeks: *M* = −20.446±*SD* 4.450; 8 weeks: *M* = −19.571±*SD* 4.399; 10 weeks: *M* = −20.982±*SD* 4.459; 12 weeks: *M* = −19.107±*SD* 4.557)(*p* > 0.05).

Under ASMR insensitive and semantic dialog conditions, compared with 2 weeks (*M* = 1.750±*SD* 1.261) and 4 weeks (*M* = ‐24.393±*SD* 2.963), the level of state anxiety after 6 weeks of training (*M* = −34.625±*SD* 3.534) decreased significantly (*p* < 0.01). While the decreased degree of state anxiety level during the following weeks of training was not significantly different from that after 6 weeks (8 weeks: *M* = −34.411±*SD* 3.359; 10 weeks: *M* = −34.196±*SD* 3.360; 12 weeks: *M* = −34.607±*SD* 3.464)(*p* > 0.05).

**FIGURE 3:**
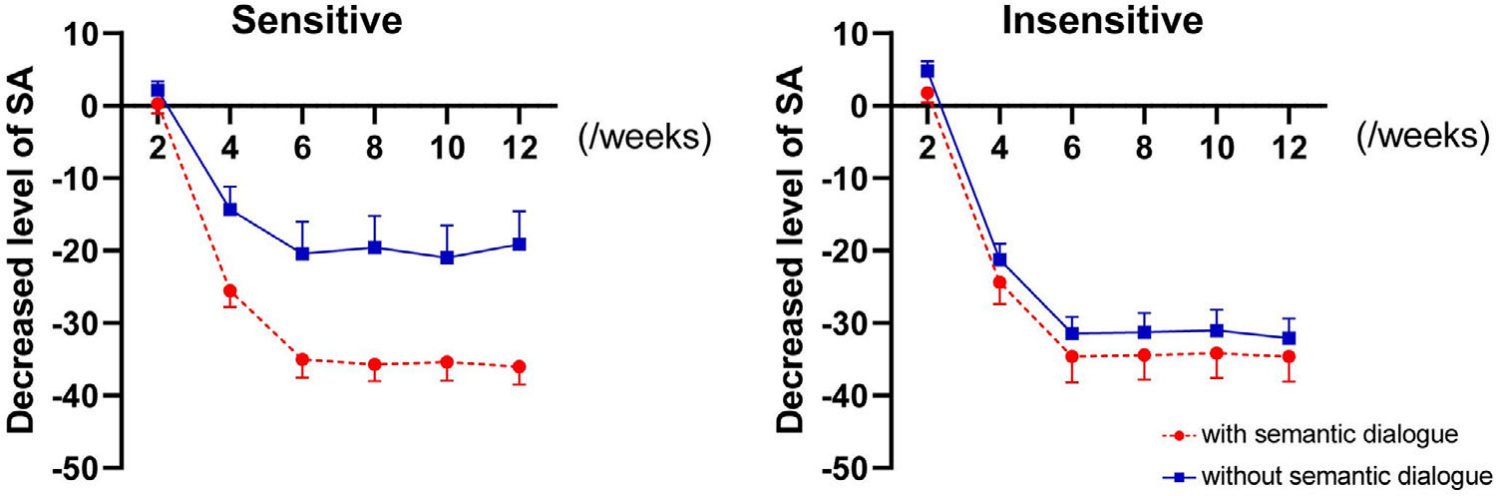
Intragroup three‐factor ANOVA with repeated measurement on state anxiety (SA) (*M*± *SE*): ASMR sensitivity, ASMR semantic dialog and ASMR training period had significant interaction (*p* < 0.05). Under ASMR sensitive and no semantic dialog condition, it firstly dropped significantly (*p* < 0.05) during 4‐weeks training and then leveled off. While under other conditions, the turning point was after 6 weeks of training (*p* < 0.01)

Under ASMR insensitive and no semantic dialog conditions, compared with 2 weeks (*M* = 5.018±*SD* 1.492) and 4 weeks (*M* = −20.911±*SD* 2.556), the level of state anxiety after 6 weeks of training (*M* = −31.196±*SD* 2.596) decreased significantly (*p* < 0.01). While the decreased degree of state anxiety level during the following weeks of training was not significantly different from that after 6 weeks (8 weeks: *M* = −30.946±*SD* 3.044; 10 weeks: *M* = −30.714±*SD* 3.284; 12 weeks: *M* = −31.821±*SD* 3.085)(*p* > 0.05). (See Table 3 and Figure 3).

**TABLE 3:** Three‐factor repeated‐measure ANOVA on state anxiety: ASMR sensitivity, semantic dialog and training period

	*SS*	*df*	*MS*	*F*	*η^2^*	1*‐α*
sensitivity	1288.583	1	1288.583	2.165	0.143	0.276
Error (sensitivity)	7737.870	13	595.221			
semantic dialog	4665.190	1	4665.190	6.080*	0.319	0.626
Error (semantic dialog)	9975.398	13	767.338			
training period	47053.103	1.566	30041.276	302.092**	0.959	1.000
Error (training period)	2024.850	20.362	99.444			
sensitivity^*^semantic dialog	1435.507	1	1435.507	2.481	0.160	0.309
Error (sensitivity^*^semantic dialog)	7523.228	13	578.710			
sensitivity^*^training period	699.365	1.579	442.784	4.008*	0.236	0.587
Error (sensitivity^*^training period)	2268.525	20.533	110.481			
semantic dialog^*^training period	450.705	1.368	329.545	2.601	0.167	0.381
Error (semantic dialog^*^training period)	2252.238	17.780	126.676			
sensitivity^*^semantic dialog^*^training period	464.924	1.730	268.667	3.677*	0.220	0.578
Error (sensitivity^*^semantic dialog^*^training period)	1643.810	22.496	73.070			
Total	89483.296	129.414				

*Notes*. *SS*, sum of squares (type III); *MS*, mean square; *η* ^2^, effect size; 1‐*α*, power of test

^a^ calculate using Alpha = .05.

* *p* < 0.05,

** *p* < 0.01.

## 3 DISCUSSION

To accurately explore the effects of ASMR videos on the reduction of psychological cravings and anxiety among forced abstainers, this study adopted a three‐factor mixed design of 2 (ASMR training/no training) × 2 (ASMR sensitive/nonsensitive) × 2 (ASMR with semantic dialog/without semantic dialog) to conduct a controled training experiment with 122 participants depending on new drugs in a female drug detoxification center in China.

The most important result of this study was that after the training, while the Acc of the experimental group in the addiction Stroop task did not change significantly both within and between groups, the Rt was significantly lower than that of the control group. This indicates that the attentional bias of the participants of the experimental group for color‐related cues reflected by the words was higher than that for drug‐related cues. It has been confirmed that drug Stroop effect is prevalent in drug addiction, and this effect is positively correlated with relapse rate (Kennedy et al., 2014; Marhe et al., 2013). Drug Stroop effect not only indicates poor control inhibition ability, but also attentional bias to drug‐related cues. Many studies have shown that attentional bias is positively correlated with subjective craving, and its validity is even higher than that of the subjective report questionnaire (Liang et al., 2019; Ramirez et al., 2015; Waters et al., 2014). Therefore, attentional bias can be used as a behavioral indicator of subjective craving.

ASMR reduces the attentional bias of drug addicts to drug‐related cues, which indicates that ASMR has a benign effect on the regulation of “psychological cravings” of drug addicts in compulsory isolation in this study. In addition, in this study, the SAI score of the experimental group was significantly reduced after 4 weeks of the training period compared with that before the training, indicating that ASMR also played a benign role in regulating the SA of the people with severe abstinence. Although it was statistically shown that ASMR effectively reduced the state anxiety of the participants, considering the subjectivity of the “questionnaire report,” the practice effect of the design before and after the test, as well as the influence of psychological comfort and other factors, the significant effect may be exaggerated. In general, ASMR is a unique form of relaxation that uses auditory and visual stimuli to bring about a calm state of mind and tingling sensations, like mindfulness (Seifzadeh et al., 2021). Some studies have shown that ASMR has the same characteristic factors as mindfulness training (Fredborg et al., 2018). It is known that mindfulness training is one of the most effective psychotherapies for addiction, and this suggests that the high correlation between ASMR and mindfulness reveals the potential for complementary psychotherapy, which may be just as effective at reducing drug cravings and anxiety as mindfulness.

Interestingly, the interaction between sensitivity, semantic dialog, and training period on state anxiety was significant. In general, the anxiety of drug addicts decreased gradually and then leveled off with the training periods; however, the changes of state anxiety in sensitive and nonsensitive participants were different in different training periods. This suggests that the content of ASMR should be measured according to the sensitivity of the audience. In recent years, ASMR has been widely used in stress management for its ability to trigger psychologically pleasurable responses (Barratt and Davis, 2015, 2017; Lee et al., 2019; Poerio et al., 2018). However, ASMR training rarely selects the type of video (for example, whether it has semantic dialog) based on the type of audience (such as sensitivity). In fact, there are differences in brain activity among spontaneous perceptual participants who are sensitive to different triggers (Smith et al., 2020). The implication for us is that in future studies, video types can be selected according to the sensitive types of the participants.

It is important to note that ASMR sensitivity/non‐sensitivity were no significant main effect on state anxiety, which may reflect the universality of ASMR or may be due to the subjective reporting used in the measurement of ASMR sensitivity. Subjective report will be affected by individual judgment criteria and social credit to some extent. Apart from subjective reports, cognitive experiments and neurophysiological instruments measured anxiety and sensitivity only indirectly. In future studies, more indicators will be considered, such as attention, EEG, and respiratory rate, to assist.

In this study, both psychological cravings and anxiety were reduced in MA female abstainers through the ASMR intervention. As shown earlier, a large number of studies have shown a positive correlation between self‐reported craving and anxiety in MA abstainers. It is known that weakened attentional bias to drug‐related cues in withdrawal clients implies a decrease in psychological cravings, and some studies have also indicated that people's weakened attentional bias to threat reduces anxiety (Bar‐Haim et al., 2007; Carmona et al., 2015; Dudeney et al., 2015; Jasper and Witthaft, 2011; Sagliano et al., 2014). So what is the link between cognitive neurophysiological mechanisms of attentional bias to drug‐related cues and response to threatening stimuli? We suggest that in‐depth studies are warranted in order to further confirm this association.

Furthermore, in terms of participant selection, this study included a sample of female forced abstainers who were all dependent on new drugs and from a single region. Thus, it lacked representativeness in terms of region, gender, and addiction type, and the training length and number of sessions were also limited. Also, in the measurement of attentional bias, the addiction Stroop task uses words of different colors as experimental materials. Thus, there may be differences in the performance of participants according to their education level; more consideration can be given to point detection paradigms using drug‐related pictures as stimulus clues. In addition, the materials in the ASMR video library used in this study were mainly created by foreign creators. Different languages, themes, and cultures may also affect the ASMR effects. In consequence, the work of creating localized ASMR materials is urgently required.

## 4 CONCLUSION

This study provides a theoretical basis for the role of ASMR in regulating psychological cravings and anxiety in forced abstainers and yielded the following findings: (1) ASMR had a positive effect on the mental health of forced abstainers; (2) the ASMR training reduced forced abstainers’ attentional bias to drug‐related clues; and (3) after 1 month of the training, the effect of ASMR in reducing SA became significant.

## CONFLICT OF INTEREST

The authors declare that the research was conducted in the absence of any commercial or financial relationships that could be construed as a potential conflict of interest.

## AUTHOR CONTRIBUTIONS

MQH, HLL, SQH and QZ developed the original idea and the protocol, drafted of the manuscript. MQH, HLL and YYQ abstracted and analyzed data, improved the manuscript. SSW, YTJ, LY and XY contributed to the critical revision of the manuscript for important intellectual content.

### PEER REVIEW

The peer review history for this article is available at https://publons.com/publon/10.1002/brb3.2636.

## Supporting information

Appendix A Clinical characteristic information

Appendix B Series with semantic dialogue

Appendix C Series without semantic dialogue

Appendix D Chinese phrase in Stroop

## Data Availability

The data that support the findings of this study are openly available in https://www.researchgate.net/profile/Qiang‐Zhou‐13, named “data set”
