# Supplementary material for: Reduction of psychological cravings and anxiety in women compulsorily isolated for detoxification using autonomous sensory meridian response (ASMR)
Source: Brain Behav. 2022 Jun 8;12(7):e2636. doi: 10.1002/brb3.2636 (PMC9304838; doi:10.1002/brb3.2636)
Supplement: Supplementary file 1 — Appendix A Clinical characteristic information [file BRB3-12-e2636-s002.docx]

**Appendix A**

**Clinical characteristic information**

| Insensitive participants (*n* = 64) | | | | | | |
| --- | --- | --- | --- | --- | --- | --- |
| Whether or not you have HIV?  -Uninfected.  Medical history?  -Denied the history of major diseases and surgical trauma, denied the history of blood transfusion and selling blood, denied the history of swallowing foreign bodies. | | | | | | |
| Num | Period of management | Date of first drug use | Method of drug use | Kind of drug | Type of drug | Drug use fixed number of year |
| 220180601 | Regression guidance period | / | burning inhale | new-type drug | methamphetamine | / |
| 220180614 | Regression guidance period | 2014 | burning inhale | new-type drug | methamphetamine | Less than 1-5 years |
| 220180617 | Regression guidance period | 2014 | burning inhale | new-type drug | methamphetamine | Less than 1-5 years |
| 220180661 | Regression guidance period | 2015 | burning inhale | new-type drug | methamphetamine | Less than 1-5 years |
| 220190053 | Regression guidance period | 2015 | burning inhale | new-type drug | methamphetamine | Less than 1-5 years |
| 220190077 | Regression guidance period | 2016 | burning inhale | new-type drug | methamphetamine | Less than 1-5 years |
| 220190112 | Regression guidance period | 2011 | burning inhale | new-type drug | methamphetamine | Less than 5-10 years |
| 220190146 | Regression guidance period | 2011 | burning inhale | new-type drug | methamphetamine | Less than 5-10 years |
| 220190160 | Regression guidance period | 2018 | burning inhale | new-type drug | methamphetamine | Less than 1-5 years |
| 220190209 | Regression guidance period | 2017 | burning inhale | new-type drug | methamphetamine | Less than 1-5 years |
| 220190210 | Regression guidance period | 2018 | burning inhale | new-type drug | methamphetamine | Less than 1 years |
| 220190217 | Regression guidance period | 2017 | burning inhale | new-type drug | methamphetamine | Less than 1-5 years |
| 220190271 | Regression guidance period | 2008 | burning inhale | new-type drug | methamphetamine | Less than 10-15 years |
| 220190278 | Regression guidance period | 2008 | burning inhale | new-type drug | methamphetamine | Less than 10-15 years |
| 220190283 | Regression guidance period | 2008 | burning inhale | new-type drug | methamphetamine | Less than 10-15 years |
| 220190290 | Regression guidance period | 2013 | burning inhale | new-type drug | methamphetamine | Less than 5-10 years |
| 220190295 | Regression guidance period | 2010 | burning inhale | new-type drug | methamphetamine | Less than 5-10 years |
| 220190296 | Regression guidance period | 2017 | burning inhale | new-type drug | methamphetamine | Less than 1-5 years |
| 220190299 | Regression guidance period | 2015 | burning inhale | new-type drug | methamphetamine | Less than 1-5 years |
| 220190303 | Regression guidance period | 2015 | burning inhale | new-type drug | methamphetamine | Less than 1-5 years |
| 220190315 | Regression guidance period | 2017 | burning inhale | new-type drug | methamphetamine | Less than 1-5 years |
| 220190334 | Regression guidance period | 2015 | burning inhale | new-type drug | methamphetamine | Less than 1-5 years |
| 220190336 | Regression guidance period | 2016 | burning inhale | new-type drug | methamphetamine | Less than 1-5 years |
| 220190343 | Convalescent period | 2012 | snuffed inhale | new-type drug | Ketamine | Less than 5-10 years |
| 220190364 | Regression guidance period | 2014 | burning inhale | new-type drug | Undefined,Ketamine | Less than 5-10 years |
| 220190380 | Convalescent period | 2017 | burning inhale | new-type drug | methamphetamine | Less than 1-5 years |
| 220190389 | Regression guidance period | 2015 | burning inhale | new-type drug | methamphetamine | Less than 1-5 years |
| 220190390 | Regression guidance period | 2013 | burning inhale | new-type drug | methamphetamine | Less than 5-10 years |
| 220190394 | Regression guidance period | 2015 | burning inhale | new-type drug | methamphetamine | Less than 1-5 years |
| 220190400 | Regression guidance period | / | burning inhale | new-type drug | methamphetamine | / |
| 220190403 | Convalescent period | 2015 | burning inhale | new-type drug | methamphetamine | Less than 1-5 years |
| 220190405 | Convalescent period | 2016 | burning inhale | new-type drug | methamphetamine | Less than 1-5 years |
| 220190406 | Convalescent period | 2017.5 | burning inhale | new-type drug | methamphetamine | Less than 1-5 years |
| 220190411 | Regression guidance period | 2016 | burning inhale | new-type drug | methamphetamine | Less than 1-5 years |
| 220190420 | Regression guidance period | 2009 | burning inhale | new-type drug | methamphetamine | Less than 10-15 years |
| 220190434 | Regression guidance period | 2010 | burning inhale | new-type drug | methamphetamine | Less than 10-15 years |
| 220190438 | Convalescent period | 2014 | burning inhale | new-type drug | methamphetamine | Less than 5-10 years |
| 220190439 | Convalescent period | 2015 | snuffed inhale | new-type drug | Ketamine | Less than 1-5 years |
| 220190444 | Regression guidance period | 2010 | burning inhale | new-type drug | methamphetamine | Less than 5-10 years |
| 220190448 | Convalescent period | 2014 | burning inhale | new-type drug | methamphetamine | Less than 5-10 years |
| 220190450 | Convalescent period | 2018 | burning inhale | new-type drug | methamphetamine | Less than 1-5 years |
| 220190453 | Regression guidance period | 2014 | burning inhale | new-type drug | methamphetamine | Less than 5-10 years |
| 220190454 | Regression guidance period | 2017 | burning inhale | new-type drug | methamphetamine | Less than 1-5 years |
| 220190455 | Convalescent period | 2017 | burning inhale | new-type drug | methamphetamine | Less than 1-5 years |
| 220190462 | Convalescent period | 2012 | burning inhale | new-type drug | methamphetamine | Less than 5-10 years |
| 220190466 | Regression guidance period | 2017 | burning inhale | new-type drug | methamphetamine | Less than 1-5 years |
| 220190468 | Regression guidance period | 2017 | burning inhale | new-type drug | methamphetamine | Less than 1-5 years |
| 220190469 | Regression guidance period | 2017 | snuffed inhale | new-type drug | Ketamine | Less than 1-5 years |
| 220190473 | Regression guidance period | 2008 | burning inhale | new-type drug | methamphetamine | Less than 10-15 years |
| 220190475 | Convalescent period | 2016 | burning inhale | new-type drug | methamphetamine | Less than 1-5 years |
| 220190484 | Regression guidance period | 2017 | burning inhale | new-type drug | methamphetamine | Less than 1-5 years |
| 220190488 | Regression guidance period | 1996 | burning inhale | new-type drug | methamphetamine | More than 15 years |
| 220190490 | Regression guidance period | 2013 | burning inhale | new-type drug | methamphetamine | Less than 5-10 years |
| 220190496 | Regression guidance period | 2005 | burning inhale | new-type drug | methamphetamine | Less than 10-15 years |
| 220200002 | Convalescent period | 2017 | burning inhale | new-type drug | methamphetamine | Less than 1-5 years |
| 220200003 | Convalescent period | 2016 | burning inhale | new-type drug | methamphetamine | Less than 1-5 years |
| 220200004 | Convalescent period | 2016 | burning inhale | new-type drug | methamphetamine | Less than 1-5 years |
| 220200006 | Convalescent period | 2012 | burning inhale | new-type drug | methamphetamine | Less than 5-10 years |
| 220200011 | Convalescent period | 2017 | burning inhale | new-type drug | methamphetamine | Less than 1-5 years |
| 220200015 | Convalescent period | 2017 | burning inhale | new-type drug | methamphetamine | Less than 1-5 years |
| 220200024 | Convalescent period | 2010 | burning inhale | new-type drug | methamphetamine | Less than 10-15 years |
| 220200026 | Convalescent period | 2017 | burning inhale | new-type drug | methamphetamine | Less than 10-15 years |
| 220200032 | Convalescent period | 2006 | burning inhale | new-type drug | methamphetamine | Less than 10-15 years |
| 220200035 | Convalescent period | 2012 | burning inhale | new-type drug | methamphetamine | Less than 5-10 years |

| Sensitive participants (*n* = 64) | | | | | | |
| --- | --- | --- | --- | --- | --- | --- |
| Whether or not you have HIV?  -Uninfected.  Medical history?  -Denied the history of major diseases and surgical trauma, denied the history of blood transfusion and selling blood, denied the history of swallowing foreign bodies. | | | | | | |
| 220180566 | Regression guidance period | 2015 | burning inhale | new-type drug | methamphetamine | Less than 1-5 years |
| 220180595 | Regression guidance period | 2015 | burning inhale | new-type drug | methamphetamine | Less than 1-5 years |
| 220180596 | Regression guidance period | / | burning inhale | new-type drug | methamphetamine | Less than 1-5 years |
| 220180615 | Regression guidance period | 2014 | burning inhale | new-type drug | methamphetamine | Less than 1-5 years |
| 220190026 | Regression guidance period | 2015 | burning inhale | new-type drug | methamphetamine | Less than 1-5 years |
| 220190082 | Regression guidance period | 2013 | burning inhale | new-type drug | methamphetamine | Less than 1-5 years |
| 220190084 | Regression guidance period | 2012 | burning inhale | new-type drug | methamphetamine | Less than 1-5 years |
| 220190086 | Regression guidance period | 2006 | burning inhale | new-type drug | methamphetamine | Less than 10-15 years |
| 220190118 | Regression guidance period | 2015 | burning inhale | new-type drug | methamphetamine | Less than 1-5 years |
| 220190140 | Regression guidance period | 2015 | burning inhale | new-type drug | methamphetamine | Less than 1-5 years |
| 220190141 | Regression guidance period | 2013 | burning inhale | new-type drug | methamphetamine | Less than 5-10 years |
| 220190184 | Regression guidance period | 2008 | burning inhale | new-type drug | methamphetamine | Less than 10-15 years |
| 220190223 | Regression guidance period | 2008 | burning inhale | new-type drug | methamphetamine | Less than 10-15 years |
| 220190224 | Regression guidance period | 2012 | burning inhale | new-type drug | methamphetamine | Less than 5-10 years |
| 220190225 | Regression guidance period | 2016 | burning inhale | new-type drug | methamphetamine | Less than 1-5 years |
| 220190227 | Regression guidance period | 2000 | burning inhale | new-type drug | methamphetamine | More than 15 years |
| 220190239 | Regression guidance period | 2018 | burning inhale | new-type drug | methamphetamine | Less than 1-5 years |
| 220190240 | Regression guidance period | 2016 | burning inhale | new-type drug | methamphetamine | Less than 1-5 years |
| 220190241 | Regression guidance period | 2010 | burning inhale | new-type drug | methamphetamine | Less than 5-10 years |
| 220190243 | Convalescent period | 2015 | burning inhale | new-type drug | methamphetamine | Less than 1-5 years |
| 220190244 | Regression guidance period | 2013 | burning inhale | new-type drug | methamphetamine | Less than 5-10 years |
| 220190248 | Regression guidance period | 2011 | burning inhale | new-type drug | methamphetamine | Less than 5-10 years |
| 220190255 | Regression guidance period | 2004 | burning inhale | new-type drug | methamphetamine | More than 15 years |
| 220190279 | Regression guidance period | 2014 | burning inhale | new-type drug | methamphetamine | Less than 5-10 years |
| 220190282 | Regression guidance period | 2005 | burning inhale | new-type drug | methamphetamine | Less than 10-15 years |
| 220190302 | Regression guidance period | 2008 | burning inhale | new-type drug | methamphetamine | Less than 10-15 years |
| 220190304 | Regression guidance period | 2011 | burning inhale | new-type drug | methamphetamine | Less than 5-10 years |
| 220190305 | Regression guidance period | 2006 | burning inhale | new-type drug | methamphetamine | Less than 10-15 years |
| 220190308 | Regression guidance period | 2008 | burning inhale | new-type drug | methamphetamine | Less than 10-15 years |
| 220190312 | Regression guidance period | 2014 | burning inhale | new-type drug | methamphetamine | Less than 5-10 years |
| 220190317 | Regression guidance period | 2010 | burning inhale | new-type drug | methamphetamine | Less than 5-10 years |
| 220190318 | Regression guidance period | 2007 | burning inhale | new-type drug | methamphetamine | Less than 10-15 years |
| 220190323 | Regression guidance period | 2014 | burning inhale | new-type drug | methamphetamine | Less than 5-10 years |
| 220190325 | Convalescent period | 1998 | burning inhale | new-type drug | methamphetamine | More than 15 years |
| 220190337 | Regression guidance period | 2015 | burning inhale | new-type drug | methamphetamine | Less than 1-5 years |
| 220190345 | Convalescent period | 2018 | burning inhale | new-type drug | methamphetamine | Less than 1-5 years |
| 220190365 | Regression guidance period | 2013 | burning inhale | new-type drug | methamphetamine | Less than 5-10 years |
| 220190369 | Convalescent period | 2017 | burning inhale | new-type drug | methamphetamine | Less than 1-5 years |
| 220190393 | Regression guidance period | 2014 | burning inhale | new-type drug | methamphetamine | Less than 5-10 years |
| 220190395 | Regression guidance period | 2016 | burning inhale | new-type drug | methamphetamine | Less than 1-5 years |
| 220190410 | Regression guidance period | 2005 | burning inhale | new-type drug | methamphetamine | Less than 10-15 years |
| 220190424 | Regression guidance period | 1995 | burning inhale | new-type drug | methamphetamine | More than 15 years |
| 220190451 | Convalescent period | 2015 | burning inhale | new-type drug | methamphetamine | Less than 1-5 years |
| 220190452 | Convalescent period | 2013 | burning inhale | new-type drug | methamphetamine | Less than 5-10 years |
| 220190457 | Convalescent period | 2016 | burning inhale | new-type drug | methamphetamine | Less than 1-5 years |
| 220190459 | Convalescent period | 2014 | burning inhale | new-type drug | methamphetamine | Less than 5-10 years |
| 220190460 | Convalescent period | 2014 | burning inhale | new-type drug | methamphetamine | Less than 5-10 years |
| 220190470 | Convalescent period | 2015 | burning inhale | new-type drug | methamphetamine | Less than 1-5 years |
| 220190471 | Regression guidance period | 2013 | burning inhale | new-type drug | methamphetamine | Less than 5-10 years |
| 220190481 | Regression guidance period | 2015 | burning inhale | new-type drug | methamphetamine | Less than 1-5 years |
| 220190485 | Regression guidance period | 2008 | burning inhale | new-type drug | methamphetamine | Less than 10-15 years |
| 220190487 | Regression guidance period | 1992 | burning inhale | new-type drug | methamphetamine | More than 15 years |
| 220190491 | Regression guidance period | 2011 | burning inhale | new-type drug | methamphetamine | Less than 5-10 years |
| 220190495 | Regression guidance period | 2014 | burning inhale | new-type drug | methamphetamine | Less than 5-10 years |
| 220190497 | Regression guidance period | 1999 | burning inhale | new-type drug | methamphetamine | More than 15 years |
| 220190498 | Regression guidance period | 2016 | burning inhale | new-type drug | methamphetamine | Less than 5-10 years |
| 220190502 | Regression guidance period | 1998 | burning inhale | new-type drug | methamphetamine | More than 15 years |
| 220190505 | Convalescent period | 1998 | burning inhale | new-type drug | methamphetamine | More than 15 years |
| 220190508 | Convalescent period | 2016 | burning inhale | new-type drug | methamphetamine | Less than 1-5 years |
| 220190509 | Convalescent period | 2009 | burning inhale | new-type drug | methamphetamine | Less than 10-15 years |
| 220200001 | Physiological detoxification period | 2011 | burning inhale | new-type drug | methamphetamine | Less than 5-10 years |
| 220200020 | Convalescent period | 2014 | burning inhale | new-type drug | methamphetamine | Less than 5-10 years |
| 220200021 | Convalescent period | 2013 | burning inhale | new-type drug | methamphetamine | Less than 5-10 years |
| 220200028 | Regression guidance period | 2015 | burning inhale | new-type drug | methamphetamine | Less than 1-5 years |
