# Supplementary material for: Reduction of psychological cravings and anxiety in women compulsorily isolated for detoxification using autonomous sensory meridian response (ASMR)
Source: Brain Behav. 2022 Jun 8;12(7):e2636. doi: 10.1002/brb3.2636 (PMC9304838; doi:10.1002/brb3.2636)
Supplement: Supplementary file 2 — Appendix B Series with semantic dialogue [file BRB3-12-e2636-s001.docx]

**Appendix B**

**Series with semantic dialogue**

| Num | Mov | Title | Sex | Dialogue | Intensity | Screenshot |
| --- | --- | --- | --- | --- | --- | --- |
| 1 | 01.  mp4 | aromatherapy | female | yes | low | 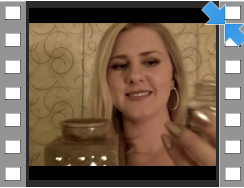   \|  \| \| --- \| |
| 2 | 02.  mp4 | facial massage | female | yes | high | 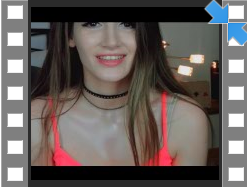   \|  \| \| --- \| |
| 3 | 35.  mp4 | tapping a little pillow | male | yes | middle | 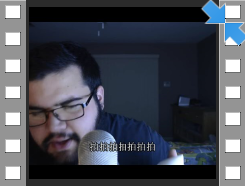 |
| 4 | 36.  mp4 | sound of a scissors | male | yes | middle | 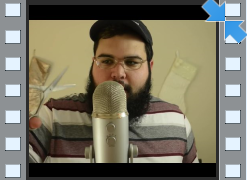 |
| 5 | 37.  mp4 | tapping a wood brick | male | yes | high | 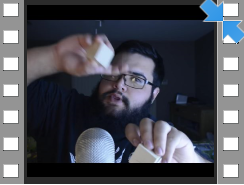 |
| 6 | 38.  mp4 | sound of mouse | male | yes | middle | 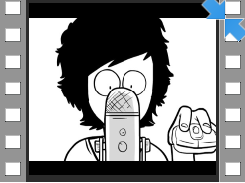 |
| 7 | 40.  mp4 | role-play of hair cutting | male | yes | middle | 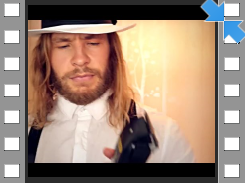 |
| 8 | 41.  mp4 | b-box | male | yes | low | 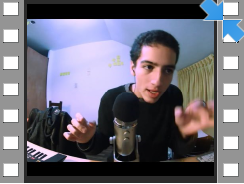 |
| 9 | 42.  mp4 | scalp massage by a man | male | yes | high | 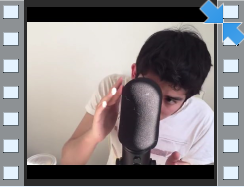 |
| 10 | 43.  mp4 | multiple whispering | male | yes | low | 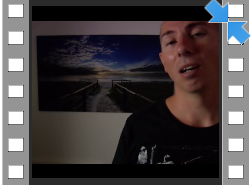 |
| 11 | 44.  mp4 | massage someone's back | male | yes | low | 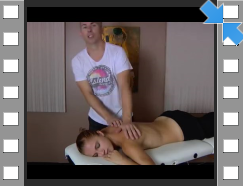 |
| 12 | 45.  mp4 | role-play of energy healing | male | yes | low | 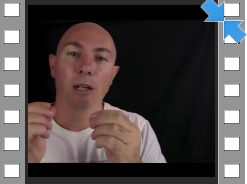 |
| 13 | 46.  mp4 | sound of scratching | male | yes | middle | 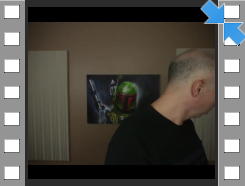 |
| 14 | 47.  mp4 | the sound of LG cracking | male | yes | middle | 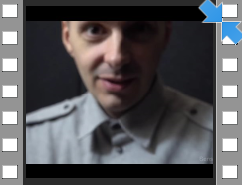 |
| 15 | 48.  mp4 | personal attention （male） | male | yes | high | 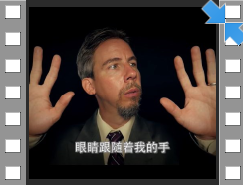 |
| 16 | 49.  mp4 | attempting to unlock | male | yes | low | 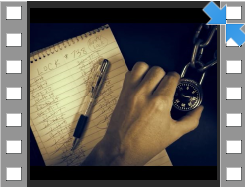 |
| 17 | 50.  mp4 | tapping a wooden comb | female | yes | low | 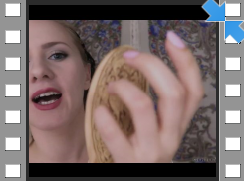 |
| 18 | 51.  mp4 | eating honeycomb | female | yes | middle | 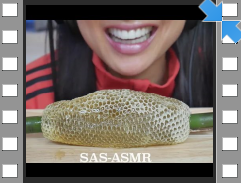 |
| 19 | 52.  mp4 | cleaning both ears at the same time | female | yes | high | 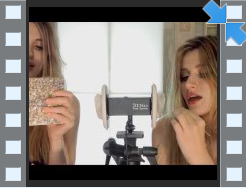 |
| 20 | 53.  mp4 | whispering | female | yes | middle | 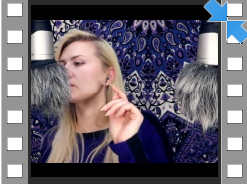 |
| 21 | 54.  mp4 | whispering and personal attention | female | yes | low | 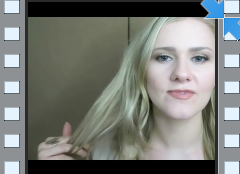 |
| 22 | 55.  mp4 | squeeze nose pore | female | yes | middle | 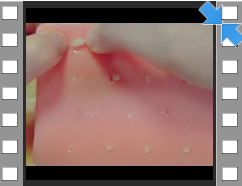 |
| 23 | 56.  mp4 | trigger words and ear cleaning | female | yes | high | 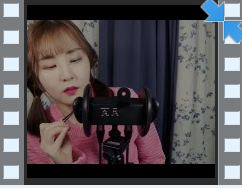 |
| 24 | 57.  mp4 | role-play of makeup | female | yes | high | 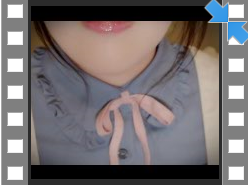 |
| 25 | 58.  mp4 | role-play of washing your hair | female | yes | middle | 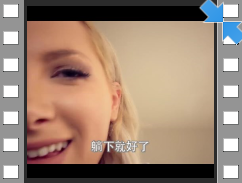 |
| 26 | 59.  mp4 | role-play of taking off your make-up | female | yes | middle | 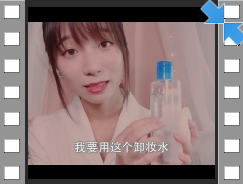 |
| 27 | 60.  mp4 | cleaning your ear by a girl | female | yes | high | 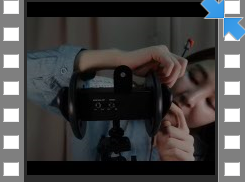 |
| 28 | 61.  mp4 | personal attention and relaxing | female | yes | low | 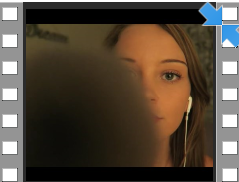 |
| 29 | 62.  mp4 | massage your temples | female | yes | low | 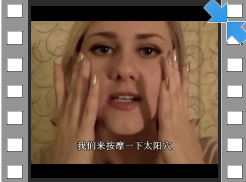 |
