# Supplementary material for: Reduction of psychological cravings and anxiety in women compulsorily isolated for detoxification using autonomous sensory meridian response (ASMR)
Source: Brain Behav. 2022 Jun 8;12(7):e2636. doi: 10.1002/brb3.2636 (PMC9304838; doi:10.1002/brb3.2636)
Supplement: Supplementary file 3 — Appendix C Series without semantic dialogue [file BRB3-12-e2636-s003.docx]

**Appendix C**

**Series without semantic dialogue**

| Num | Mov | Title | Sex | Dialogue | Intensity | Screenshot |
| --- | --- | --- | --- | --- | --- | --- |
| 1 | 03.  mp4 | ear licking | female | no | high | 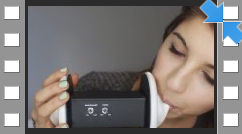   \|  \| \| --- \| |
| 2 | 06.  mp4 | sound of tongue | female | no | middle | 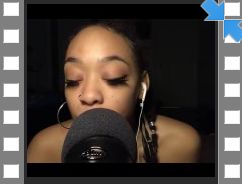 |
| 3 | 07.  mp4 | eating Salmon and octopus | female | no | high | 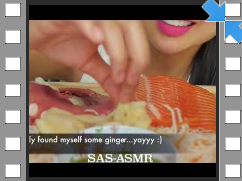 |
| 4 | 08.  mp4 | combing your hair | female | no | low | 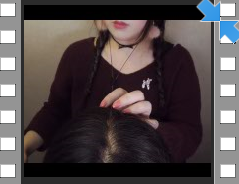 |
| 5 | 09.  mp4 | sound of rain | female | no | low | 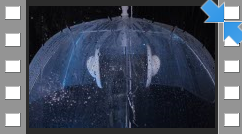 |
| 6 | 10.  mp4 | tapping and scratching | female | no | middle | 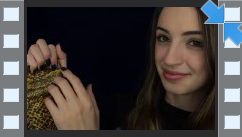 |
| 7 | 11.  mp4 | eat chill | female | no | middle | 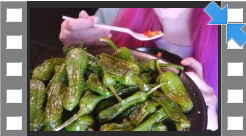 |
| 8 | 12.  mp4 | facial cosmetic facial cosmetic | female | no | middle | 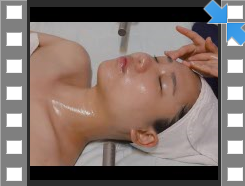 |
| 9 | 13.  mp4 | remove thorn | female | no | low | 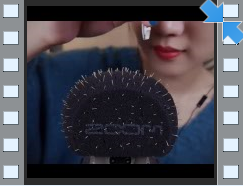 |
| 10 | 14.  mp4 | ear massage | female | no | high | 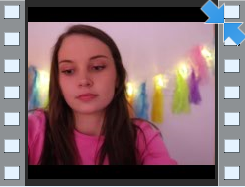 |
| 11 | 15.  mp4 | touching your face | female | no | low | 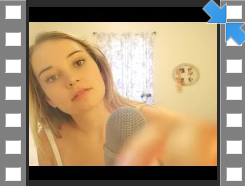 |
| 12 | 16.  mp4 | scalp massage | female | no | low | 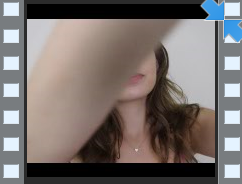 |
| 13 | 17.  mp4 | touching your face and mouth sound | female | no | middle | 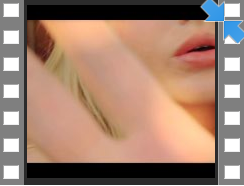 |
| 14 | 18.  mp4 | cleaning ear by Cotton swab | female | no | high | 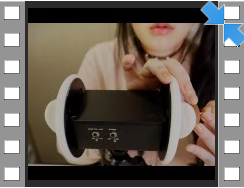 |
| 15 | 19.  mp4 | eating caviar and sea grape | female | no | high | 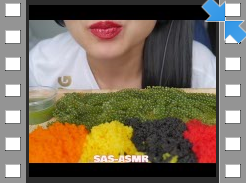 |
| 16 | 20.  mp4 | grind salt | male | no | high | 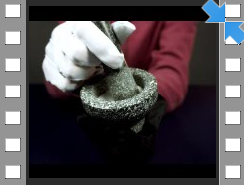 |
| 17 | 21.  mp4 | clean in your earwax by a man | male | no | high | 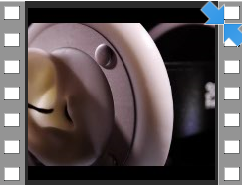 |
| 18 | 22.  mp4 | mixing beads and glue | male | no | low | 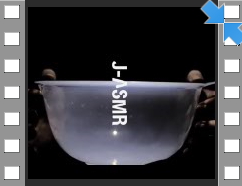 |
| 19 | 23.  mp4 | brushing your ear by a soft brush | male | no | high | 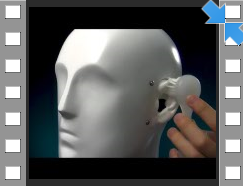 |
| 20 | 24.  mp4 | mixing slime beads | male | no | low | 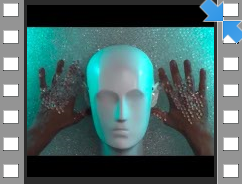 |
| 21 | 25.  mp4 | sound of soda water | male | no | high | 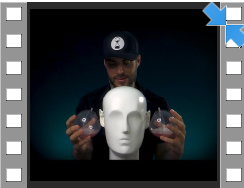 |
| 22 | 26.  mp4 | ear massage by a Silicone beauty blender | male | no | low | 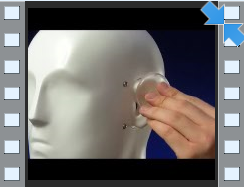 |
| 23 | 27.  mp4 | ear cleaning by a swab | male | no | middle | 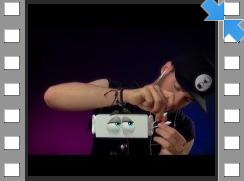 |
| 24 | 28.  mp4 | different trigger | male | no | low | 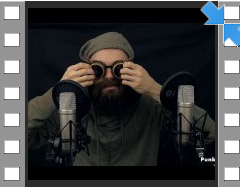 |
| 25 | 29.  mp4 | multiple mouth sound | male | no | high | 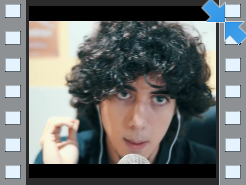 |
| 26 | 30.  mp4 | tapping glass | male | no | high | 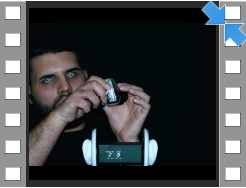 |
| 27 | 31.  mp4 | writing | male | no | middle | 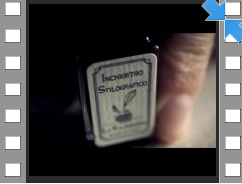 |
| 28 | 32.  mp4 | soap carving | male | no | low | 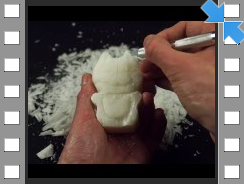 |
| 29 | 33.  mp4 | archaeological dig bone | male | no | middle | 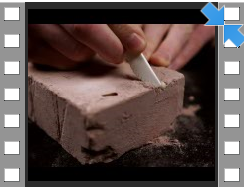 |
| 30 | 34.  mp4 | mouth sound | male | no | middle | 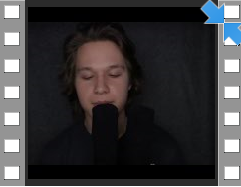 |
