# Supplementary material for: Reduction of psychological cravings and anxiety in women compulsorily isolated for detoxification using autonomous sensory meridian response (ASMR)
Source: Brain Behav. 2022 Jun 8;12(7):e2636. doi: 10.1002/brb3.2636 (PMC9304838; doi:10.1002/brb3.2636)
Supplement: Supplementary file 4 — Appendix D Chinese phrase in Stroop [file BRB3-12-e2636-s004.docx]

**Appendix D**

**Chinese phrase in Stroop**

| Number | Chinese phrase | Attribution |  | Number | Chinese phrase | Attribution |
| --- | --- | --- | --- | --- | --- | --- |
| 1 | 白菜 | neutral |  | 1 | 烫吸 | drug-related |
| 2 | 黄瓜 | neutral |  | 2 | 鼻吸 | drug-related |
| 3 | 排骨 | neutral |  | 3 | 毒品 | drug-related |
| 4 | 猪肉 | neutral |  | 4 | 白粉 | drug-related |
| 5 | 苹果 | neutral |  | 5 | 成瘾 | drug-related |
| 6 | 橘子 | neutral |  | 6 | 冰壶 | drug-related |
| 7 | 楼房 | neutral |  | 7 | 吸管 | drug-related |
| 8 | 阳台 | neutral |  | 8 | 迪厅 | drug-related |
| 9 | 椅子 | neutral |  | 9 | 会所 | drug-related |
| 10 | 桌子 | neutral |  | 10 | 罂粟 | drug-related |
| 11 | 地板 | neutral |  | 11 | 吗啡 | drug-related |
| 12 | 风扇 | neutral |  | 12 | 大麻 | drug-related |
| 13 | 厨师 | neutral |  | 13 | 黄皮 | drug-related |
| 14 | 工人 | neutral |  | 14 | 恶心 | drug-related |
| 15 | 农民 | neutral |  | 15 | 麻木 | drug-related |
| 16 | 教授 | neutral |  | 16 | 飘忽 | drug-related |
| 17 | 学生 | neutral |  | 17 | 迷幻 | drug-related |
| 18 | 尺子 | neutral |  | 18 | 兴奋 | drug-related |
| 19 | 橡皮 | neutral |  | 19 | 高潮 | drug-related |
| 20 | 铅笔 | neutral |  | 20 | 戒毒所 | drug-related |
| 21 | 黑板 | neutral |  | 21 | 金三角 | drug-related |
| 22 | 书本 | neutral |  | 22 | 银三角 | drug-related |
| 23 | 钢琴 | neutral |  | 23 | 黑三角 | drug-related |
| 24 | 古筝 | neutral |  | 24 | 金新月 | drug-related |
| 25 | 大鼓 | neutral |  | 25 | 海洛因 | drug-related |
| 26 | 唢呐 | neutral |  | 26 | 摇头丸 | drug-related |
| 27 | 笛子 | neutral |  | 27 | 古柯叶 | drug-related |
| 28 | 北京 | neutral |  | 28 | 可卡因 | drug-related |
| 29 | 杭州 | neutral |  | 29 | 塑封袋 | drug-related |
| 30 | 上海 | neutral |  | 30 | 注射器 | drug-related |
